# Protection by extra virgin olive oil against oxidative stress *in vitro* and *in vivo*. Chemical and biological studies on the health benefits due to a major component of the Mediterranean diet

**DOI:** 10.1371/journal.pone.0189341

**Published:** 2017-12-28

**Authors:** Miriam Rossi, Francesco Caruso, Lorraine Kwok, Grace Lee, Alessio Caruso, Fabio Gionfra, Elena Candelotti, Stuart L. Belli, Nora Molasky, Kathleen M. Raley-Susman, Stefano Leone, Tomáš Filipský, Daniela Tofani, Jens Pedersen, Sandra Incerpi

**Affiliations:** 1 Vassar College, Department of Chemistry, Poughkeepsie, NY, United States of America; 2 Department of Sciences, University Roma Tre, Roma, Italy; 3 Vassar College, Department of Biology, Poughkeepsie, NY, United States of America; 4 Department of Pharmacology and Toxicology in Hradec Králové, Charles University in Prague, Heyrovského, Czech Republic; 5 Department of Biology, University Tor Vergata, Rome, Italy; University of Palermo, ITALY

## Abstract

We report the results of *in vivo* studies in *Caenorhabditis elegans* nematodes in which addition of extra virgin olive oil (EVOO) to their diet significantly increased their life span with respect to the control group. Furthermore, when nematodes were exposed to the pesticide paraquat, they started to die after two days, but after the addition of EVOO to their diet, both survival percentage and lifespans of paraquat-exposed nematodes increased. Since paraquat is associated with superoxide radical production, a test for scavenging this radical was performed using cyclovoltammetry and the EVOO efficiently scavenged the superoxide. Thus, a linear correlation (y = -0.0838x +19.73, regression factor = 0.99348) was observed for superoxide presence (y) in the voltaic cell as a function of aliquot (x) additions of EVOO, 10 μL each. The originally generated supoeroxide was approximately halved after 10 aliquots (100 μL total). The superoxide scavenging ability was analyzed, theoretically, using Density Functional Theory for tyrosol and hydroxytyrosol, two components of EVOO and was also confirmed experimentally for the galvinoxyl radical, using Electron Paramagnetic Resonance (EPR) spectroscopy. The galvinoxyl signal disappeared after adding 1 μL of EVOO to the EPR cell in 10 minutes. In addition, EVOO significantly decreased the proliferation of human leukemic THP-1 cells, while it kept the proliferation at about normal levels in rat L6 myoblasts, a non-tumoral skeletal muscle cell line. The protection due to EVOO was also assessed in L6 cells and THP-1 exposed to the radical generator cumene hydroperoxide, in which cell viability was reduced. Also in this case the oxidative stress was ameliorated by EVOO, in line with results obtained with tetrazolium dye reduction assays, cell cycle analysis and reactive oxygen species measurements. We ascribe these beneficial effects to EVOO antioxidant properties and our results are in agreement with a clear health benefit of EVOO use in the Mediterranean diet.

## Introduction

The use of the fruit and oil from the olive tree (*Olea europea*) in human nutrition, health and cosmetics for the civilizations centered on the Mediterranean Sea is documented from about 6000 years ago and continues to be of economic and cultural significance. Recent studies point to the region where the olive tree was domesticated as near Syria and Turkey [1] whereas widespread distribution of the olive tree within southern Europe and northern Africa was carried out by the Romans and Phoenicians. The olive tree continues to occupy a central role in traditions and various religious rituals of populations along the Mediterranean. For example, the founding of the important Greek city-state Athens is associated with the olive tree since it was a gift from the Greek goddess Athena over 5000 years ago [2]. The importance of the olive tree and its fruit has not diminished in modern times and, besides its nutritive value, it persists as a global symbol for peace.

Although the health and medicinal facets of olive oil consumption has been widely accepted since these ancient times, it is only recently that many epidemiological and biological studies have demonstrated the positive effects on human health when the diet (“the Mediterranean diet”) includes regular consumption of extra virgin olive oil (EVOO). These include reducing oxidative damage in the body and lower incidence of cancers, improved cardiovascular health, and healthier aging [3–6]. These health benefits have been attributed to the chemical composition in EVOO. The beneficial health action from diets containing EVOO is a subject of social importance, highly investigated and sometimes open to criticisms as for the trial study PREDIMED. This study analyzed the effects of diets supplemented with EVOO on a population having major risk factors (smoking, hypertension, elevated LDL cholesterol levels, overweight or obesity) but not yet affected by coronary diseases [7]. Another trial, known as the Three-City Study included 7625 subjects also at risk for coronary diseases and concluded that, in older subjects, high olive oil consumption provided a protective role towards the risk of stroke [8].

Olive oil is unique among vegetable oils since it is obtained by mechanically squeezing the fruit. The compounds found in EVOO occur in variable proportions due to differing olive cultivar types, processing methods and geo-climatic parameters [6,9]. Olive oils are composed mainly of triglycerides of fatty acids with the monounsaturated omega-9 fatty acid, oleic acid, always being the major component (from 55–83%). EVOO also contains two essential fatty acids whose compositions are 3.5 to 21% linoleic acid (a polyunsaturated omega-6) and less than 1% linolenic acid (a polyunsaturated omega-3 fatty acid). Other important but minor components include phenolic compounds, which are thought to be responsible for the antioxidant effects, including tyrosol and hydroxytyrosol and their metabolic derivatives, vitamin E, and phytosterols. More specifically, tyrosol is an active scavenger of reactive oxygen species and increases the life span in nematodes [10–11]. It also was shown that EVOO polyphenols protect against the oxidative damage in human-colon adenocarcinoma cell lines [12].

To evaluate the effects of EVOO on biological systems, we used the model organism *Caenorhabditis elegans* and two cell lines, L6 myoblasts from rat skeletal muscle and human monocytes THP-1, non tumor and tumor cells respectively. The nematode is an excellent model organism due to its well-characterized life cycle, fully sequenced genome, and easy manipulation under controlled growth conditions. Aging in *C*. *elegans*, like humans, is influenced by a combination of genetic and environmental factors; thus, studying the mechanism and phenotypical changes associated with aging in *C*. *elegans* can inform the mechanism in humans. The phenotypical changes observed as nematodes age include morphological deterioration, increased mortality rate, and decline in locomotion, fertility, and feeding [10]. Application of dietary antioxidants to the nematodes’ growing media allows researchers to determine whether there is a protective effect against oxidative stress and its associated characteristics. In previous studies, *C*. *elegans* exhibited fewer characteristics associated with oxidative stress induced by paraquat when also exposed to one of the principle phenolic compounds in EVOO, tyrosol [10–11].

In this work we describe antioxidant activity of two EVOOs, one from Italy (Rome) and one from USA (California), using the following chemical and biological assay methods: 1) Cyclic voltammetry scavenging of superoxide radical in aprotic solvent (DMSO), and related theoretical calculation; 2) Scavenging of galvinoxyl radical using electron paramagnetic resonance (EPR) technique; 3) Effects on cell proliferation and cytoxicity in two cell cultures, L6 myoblasts and human THP-1 monocytes, measured by a tetrazolium dye (MTT) assay, reactive oxygen species (ROS) determination, and cell cycle analysis; 4) *In vivo* effects on lifespan in a *C*. *elegans* model, where EVOO was included in their diet.

## Materials and methods

### Extra virgin olive oils

The two cold-pressed EVOOs used in this study were the Corto^®^ oil from Corto Olive Co., Lodi, California 95241 and the Oro delle Donne from the Società Agricola L'Oro Delle Donne, 00047 Marino, Roma, Italy.

### Chemicals

DMSO (anhydrous, ≥99.9%), tert-butyl ammonium bromide (TBMA), [(2,2-dimethyl-6,6,7,7,8,8,8-heptafluoro-3,5-octanedionato)silver(I)] and paraquat were purchased from Sigma-Aldrich (St. Louis, MO). Roswell Park Memorial Institute medium (RPMI 1640), Dulbecco´s modified Eagle medium (DMEM), sodium pyruvate (100 mM) streptomycin (100 mg/ml), penicillin (100 U/ml), phosphate buffered saline (PBS: 10 mM Na_2_HPO_4_, 137 mM NaCl 2,7 mM KCl dissolved in 500 ml of distilled water, pH 7.4). D-glucose (5 mM), dimethyl sulfoxide (DMSO), propidium iodide, Galvinoxyl, cumene hydroperoxide, 3-(4,5-dimethylthiazol-2-yl)-2,5-diphenyltetrazolium bromide (MTT), phosphate buffered saline (PBS)- one tablet/L buffer without Calcium and Magnesium were purchased from Sigma-Aldrich; 2',7'-dichlorodihydrofluorescein diacetate (DCFH_2_-DA) was obtained from Molecular Probes (Eugene, OR). Sterile plasticware for cell culture was from Falcon Brand, San Diego (CA), fetal bovine serum was from GIBCO (Grand Island, NY).

### Organisms

The Bristol N2 (wild-type) *C*. *elegans* and the *Escherichia coli* (*E*. *coli*) OP50 strain were obtained from the *Caenorhabditis* Genetics Center (CGC). The 35 mm and 60 mm petri dishes and NGM media components were purchased from Sigma-Aldrich.

### Paraquat-induced oxidative stress assay on *C*. *elegans*

#### Maintenance of and treatment conditions

There are two biological sexes in *C*. *elegans*, self-fertilizing hermaphrodites and males, which result rarely from meiotic non dysjunction of the X chromosome. In this study, we restricted our analysis to hermaphroditic nematodes because males are very rare within typical populations of worms. Bristol (N2) wild-type nematodes were grown at 20°C on nematode growth medium (NGM) agar plates with a lawn of *E*. *coli* strain OP50 for food as described previously [13]. Small, synchronized worm cultures of 50–100 worms were prepared by placing six to ten gravid adults on a plate for several hours to lay eggs. Then, the adults were removed and these timed cultures grew to the L4 stage for experimentation. For treatments involving the Oro EVOO, the NGM was made and 500 μL of oil was dissolved in 1 mL of DMSO and then added to 100 mL of the agar solution and petri plates prepared. Paraquat was prepared in distilled water and 50μL was spread using a sterile glass rod across the surface of 2 cm petri dishes.

#### Survival and stress resistance assays

We assessed the effects of Oro oil on overall lifespan by plating ten L4 worms to each of twelve plates, half containing Oro EVOO and half without. Each day, for seven consecutive days, we assessed survivorship of the worms. Every other day, we moved the surviving worms to fresh plates (with or without the oil) in order to avoid possible interference of nematode offspring. We analyzed the percent surviving nematodes in the two conditions using a two-way ANOVA with main factors of time (day) and treatment, followed by the Bonferroni post-hoc test for multiple comparisons. We set the α top less than to 0.05 for statistical significance. A similar procedure was followed to evaluate the effects of paraquat exposure in the presence or absence of Oro EVOO on survivorship. Six replicates of ten L4 on each petri plate with or without Oro EVOO in the presence or absence of 4 mM paraquat were scored for survival over seven consecutive days and assessed for their movement and response to touch. Worms that did not move or respond to touch from the platinum wire of the worm pick were noted as “dead.” Every other day, the surviving nematodes were transferred to a new treatment plate to ensure that there would not be progeny overgrowth that could obscure survival scoring. The nematodes’ locomotion was qualitatively observed and their mortality was quantitatively scored. Data are presented as the average percent survival across the replicates. Two-way ANOVA with main factors of exposure time and oil treatment were performed, followed by Bonferroni post-hoc tests for multiple comparisons, using Prism 5.0 software (GraphPad, Inc). A p value less than 0.05 was considered statistically significant.

### Cyclic voltammetry

#### Preliminary tests

The redox potential for the O_2_/superoxide cycle is shown in S1 Fig. The main goal was to determine the potential corresponding to the oxidation of superoxide (O_2_^-•^ → O_2_ + e^-^), which is needed to fix the potential at the later used ring electrode in the rotating ring disk electrode (RRDE) system, see below. The related parameter then was -0.25V. S1 Fig was obtained using a Solartron SI 1287 electrochemical interface, which has three electrodes: reference, working and counter electrodes. The reference electrode was made in the lab using Ag/AgCl wire in solution of DMSO with tetrabutylammonium bromide (TBAB) contained in a thin glass tube. The working electrode was a BASi (Bio analytical systems Inc.) Au disk electrode (MF-2014). The counter electrode was a Pt wire. The CV run used a scan rate of 100mV/sec.

#### The rotating ring disk electrode (RRDE) system

The main experiments were performed with a rotating ring disk electrode, RRDE, (Pine Instrumentation) using a Au/Au electrode with a platinum reference electrode and a platinum counter electrode. Voltammograms from all studies were collected using Aftermath software provided by Pine Instruments. Initial tests were performed using the ferri/ferrocyanide redox reaction to ensure that the electrode was operating within theoretical parameters. The voltaic cell was prepared with 40ml of a 0.005M ferricyanide solution, a platinum counter electrode submerged in a 1M Na_2_SO_4_, and a platinum reference electrode submerged in a salt solution provided by Pine Instruments.

#### Preliminary superoxide tests

To test the superoxide/oxygen redox reaction 50ml of DMSO was transferred into a voltaic cell with a 10ml glass syringe and TBAB was added. The counter electrode (Pt wire) and reference electrode (Pt wire in a DMSO/TBAB solution) were added to the voltaic cell. The disk potential was swept from 0.1 to -2.0V to reduce oxygen to superoxide, and the ring was held at a constant potential of -0.25V to collect the superoxide molecule and oxidize them back to oxygen. The CV contains 2 lines superimposed, further described in the Results and Discussion section.

#### Water interfering with the DMSO

The disk was swept from 0.1 to -2.0 V and the ring was held constant at -0.25 V. The electrode was rotated at 250 rpm at a sweep rate of 25 mV/s. A controlled amount of distilled H_2_O (range 200 μl– 1600 μl) was then added to the solution; voltammograms were collected after each new addition of water. Voltammogram results were anomalous at the ring disc due to the spontaneous disproportionation of superoxide (O_2_^-•^ + O_2_^-•^ + 2H^+^ → O_2_ + H_2_O_2_). This well-known reaction, that is also carried out by the enzyme superoxide dismutase, depends on the availability of protons, which normally come from water. We concluded that dehydrated DMSO is absolutely needed for this experiment and used DMSO (dehydrated 99.9%).

#### Bubbling with nitrogen and oxygen

The voltaic cell was prepared with 0.1M TBAB in 50ml of DMSO. The polished Au/Au electrode was then submerged into the DMSO solution, and the counter and reference electrodes were added to the cell. A glass gas conduit was added to the cell and nitrogen gas (1 atm) was bubbled through the DMSO for five minutes. Oxygen gas (1 atm) was then bubbled through the DMSO for ten minutes. The disk was swept from 0.1V to -1.5V and the ring was held constant at -0.25V. The Au/Au electrode was then rotated at 500rpm while the sweep rate was held constant at 25mV/s. All tests were made with Corto oil.

### Computational study

The theoretical study involved calculations using software programs from Biovia San Diego, CA, USA. Density functional theory code DMol^3^ was applied to calculate energy, geometry and frequencies implemented in Materials Studio 7.0 (PC platform) [14]. We employed the double numerical polarized (DNP) basis set that includes all the occupied atomic orbitals plus a second set of valence atomic orbitals, and polarized d-valence orbitals [15], and correlation generalized gradient approximation (GGA) was applied including Becke exchange [16] plus Perdew correlation [17] (BP). All electrons were treated explicitly and the real space cutoff of 5 Å was imposed for numerical integration of the Hamiltonian matrix elements. The self-consistent-field convergence criterion was set to the root-mean-square change in the electronic density to be less than 10^−6^ electron/Å^3^. The convergence criteria applied during geometry optimization were 2.72 10^-4^eV for energy and 0.054 eV/ Å for force.

### EPR study

Samples containing 50μl solutions were drawn into glass capillaries, sealed and measured using an ESP300 (Bruker Spectrospin, Karlsruhe, Germany) equipped with high sensitivity TM110 X-band cavity [18]. EPR spectra were recorded at room temperature, using 1.0 g modulation, 10 mW microwave power and a scan time of 21 s for a 50 g spectrum. Four spectra were accumulated for each measurement in order to obtain a suitable signal to noise ratio. Comparison with an EVOO supermarket oil was also performed. Experiments with EPR were conducted with 1μl oil (1μl oil to 10 μM galvinoxyl in 100μl DMSO), whereas experiments with 0.2 μl oil were made with 1 μl oil to 500 μl DMSO containing 10 μM galvinoxyl.

### Cells in culture

L6 myoblasts from rat skeletal muscle were obtained from the American Type Culture Collection (ATCC, Rockville, MD). Cells were seeded in 75 cm^2^ tissue culture flasks and grown in Dulbecco’s modified Eagle’s medium (DMEM) containing 4.5 g/l glucose, supplemented with 10% heat-inactivated fetal bovine serum, 100 μg/ml streptomycin, and 100 U/ml penicillin, in an atmosphere of 5% CO_2_ at 37°C. Cells reached confluency after 5 days (approximately 6.0 x 10^6^ cells/flask) and were kept in culture as myoblasts by continuous passages at preconfluent stages as previously reported [18]. Human leukemic monocytes, THP-1, from American Type Culture Collection (Rockville, MD, USA), were grown in suspension in RPMI-1640 medium with 10% FBS, 100 μg /ml streptomycin and 100 U/ml penicillin, in a humidified atmosphere with 5% CO_2_ a t 37°C [19–20] THP-1 monocytes were passaged twice a week by 1:4 dilutions and re-seeded; cells from passages 7–23 were used for the experiments.

The oil treatment of the cells was carried out as follows: a suspension of oil and DMSO was prepared at the time of the experiments mixing 200 μl DMSO with 1000 μl of oil. Cells, either L6 or THP-1 with the oil/DMSO suspension giving 10 μl/ml for the low dose (ld) and 50 μl/ml for the high dose (hd). By this procedure the amount of DMSO in the presence of the cells was lower than 1%. This treatment was carried out for all experiments.

#### Intracellular ROS determination

Reactive oxygen species (ROS) were measured both in L6 and THP-1 cells. The method used was a standard assay based on the intracellular fluorescent probe DCF [20]. For L6 myoblasts, the medium was discarded and cells were washed twice with 5 ml phosphate buffered saline (PBS) containing 5.0 mM glucose (PBS-glucose), at 37°C. Cells were gently scraped off with PBS-glucose at 37°C and centrifuged at 1200 rpm for 5 min, the supernatant was discarded and the pellet resuspended in PBS-glucose. The THP-1 monocytes were centrifuged at 1200 rpm for 10 min; the supernatant was discarded and cells were washed twice with 5 ml PBS-glucose at 37°C to remove the serum that might affect the action of the fluorescent probe. After the last centrifugation the pellet was resuspended in PBS-glucose and the probe DCFH2-DA was added at 10 μM final concentration. From that point onwards the protocols for the two cell types were identical. Incubation with the probe DCFH2-DA at a final concentration of 10 μM (from a stock solution of 10 mM in dimethyl sulfoxide) was carried out for 30 min in the dark at 37°C. At the end of the incubation cells were washed twice, centrifuged at 1200 rpm for 5 min, and the final cell pellet was resuspended in PBS-glucose. Before the experiments cells recovered at room temperature for 1h. ROS production was measured in a Perkin-Elmer Multilabel Counter Victor3V Wallac 1420, by evaluating the intracellular DCF fluorescence. We measured the ability of oils to buffer the reactive oxygen species (ROS) production in cells exposed to the radical generator cumene hydroperoxide (200 μM) at different times (10 min, 30 min, 1h, 3h and 24h). For the L6 myoblasts the concentration of Cumene hydroperoxide was 27.5 μM as previously reported [20]. Before addition of cumene hydroperoxide, cells were pre-incubated with oils at 37°C for 10 min, as reported earlier.

The antioxidant activity of oils was determined by the decrease in intracellular DCF fluorescence in the different experimental conditions. None of the compounds tested gave rise to fluorescence on their own. In a separate series of experiments, we ensured that DMSO concentrations had no effect on evoked responses.

#### Proliferation studies

L6 cells were seeded in 60 x 15 mm Petri dishes (1.0–1.5x10^5^ cells/dish) and grown in medium supplemented as reported in Materials and Methods. The following day the medium was discarded, and 1 ml of new medium containing different concentrations of oils were added. L6 cells were counted every 24 hours, in a Neubauer chamber, after mild trypsinization. THP-1 cells were seeded in a 24 multiwell (6.0–6.5 x10^4^ cells/well) and grown in medium supplemented as reported in Materials and Methods. THP-1 monocytes were stimulated with oils the day after seeding and counted up to 96h from seeding, after a mild resuspension, using a Neubauer chamber.

#### MTT assay

Cell viability and the possible cytotoxic effect of tested oils were assessed by the MTT assay [20]. L-6 cells were seeded in 96-wells plates at 10,000 cells/well in 200 μl DMEM containing 10% serum. The day after seeding the medium was discarded and 100 μl of new medium containing cumene (27.5 μM) and different concentrations of oils were added to each well, depending on the specific experimental condition as reported in a previous paragraph. Then MTT solution (5 mg/ml in PBS) was added at the final concentration of 10% with respect to the total volume, and incubation was carried out at 37°C for 3–4 h in the dark. During the incubation, there was a conversion of the yellow MTT to purple formazan by the mitochondrial succinate dehydrogenase of living cells. Then lysis buffer was added and further incubation at 37°C for 30 min in the dark was carried out. The lysis buffer was prepared by dissolving 40% (w/v) sodium dodecyl sulfate in deionized water, mixing an equal volume of N,N-dimethylformamide with the sodium dodecyl sulfate solution, and adjusting the pH to 4.7. Cells were then resuspended and the optical density was read with an ELISA-reader at 550–570 nm. Results are reported as the mean ± SD of 2 experiments carried out in quintuplicate.

### Cell cycle analysis

#### Flow cytometry

For cell cycle analysis, after each treatment, 1 x 106 L6 cells was washed twice with PBS, fixed dropwise with ice cold ethanol (70%) and rehydrated with PBS. DNA staining was performed by incubating cells for 30 min at 37°C in PBS containing 0.18 mg/ml propidium iodide (PI) and 0.4 mg/ml DNase-free RNase (type 1-A). Samples were acquired with a Dako Galaxy Flow Cytometer equipped with a 488 nm laser source. Cell cycle analysis was performed over 20.000 events using a FloMax v2.4e software. Doublet discrimination was performed by an electronic gate on FL3-Area vs. FL3-Height.

#### Statistical analysis

The results are reported as mean ± S.D. and analysed by either one-way Anova or two-way ANOVA analysis of variance (Anova), followed by post-hoc Bonferroni’s Multiple Comparison Test or by the Student’s *t* test; these were carried out using the Prism4 statistics program (GraphPad software). Differences were considered significant at p < 0.05.

## Results and discussion

### Computational study

**Fig 1:**
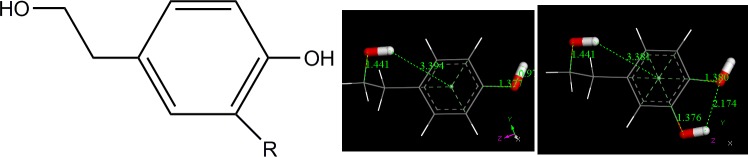
2-D formal structure of hydroxytyrosol (R: OH) and tyrosol (R: H), left. Geometry optimized hydroxytyrosol (center) and tyrosol (right) using Dmol^3^ DFT program; OH groups highlighted in thicker stick mode.

As representatives of antioxidants small molecules present in EVOO, tyrosol and hydroxytyrosol (Fig 1) were chosen to verify theoretically their capability of sequestering the superoxide radical, in a similar condition as analyzed by cyclic voltammetry in this work, that is in DMSO solvent (reaction **1**).

$$Hydroxytyrosol\;\left(Tyrosol\right)+{O_{2}}^{-•}\to Hydroxytyrosol\;{\left(Tyrosol\right)}^{•}+H{O_{2}}^{-}$$ (1)

The pendant (CH_2_)_2_OH moiety was rotated along the C(aromatic)-C(aliphatic) bond in several positions. At the minimum of energy the hydroxyl moiety protrudes towards the center of the aromatic ring (Fig 1), which is a feature seen in small aromatic hydrocarbons in their crystal structures, in contrast with larger aromatic species that tend to establish a stacking arrangement as seen in graphite. For instance, the distance between H(benzene) and a neighbouring benzene centroid is about 3 Å [21]. Hydroxytyrosol shows a H-bond between the H(hydroxyl) in position 3 towards the OH in position 4 (2.174 Å).

**Fig 2:**
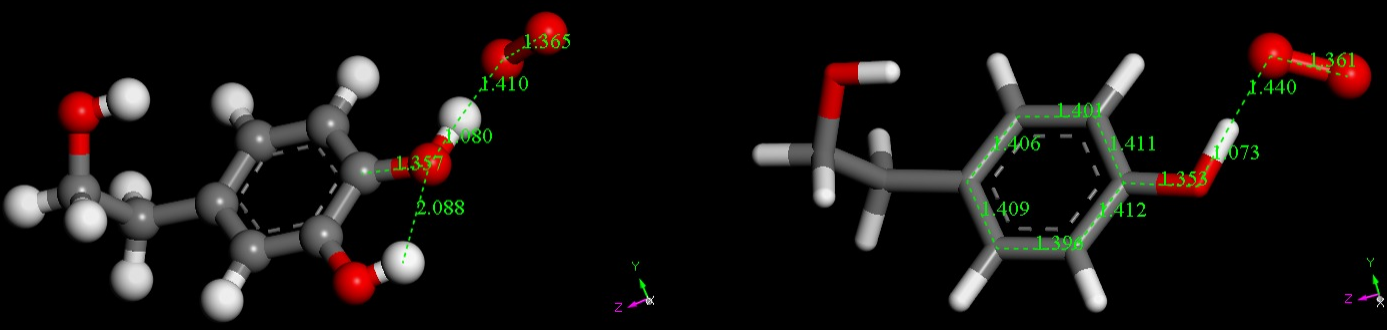
The initial state for reaction (1) between hydroxytyrosol (left) or tyrosol (right) with superoxide radical O_2_^−•^ obtained through geometry minimization. Initial separation between O(superoxide) and H was set at 2.60 Å, the sum of van der Waals radii.

Each DFT minimized compound in Fig 1 was posed for a potential approach of superoxide radical, that is, a previously geometry-minimized superoxide radical and the H(hydroxyl) in position 4 were located at van der Waals separation. The resulting geometry optimizations (Fig 2) show that for hydroxytyrosol there is strengthening of the intramolecular H-bond between the H(hydroxyl) in position 3 and adjacent O in position 4 (2.088 Å) in comparison with 2.174 Å in Fig 1, suggesting an increased negative charge on O(4). In addition, there is a slightly lengthening of OH at position 4 in hydroxytyrosol, with O-H distance of 1.080 Å, compared with 1.073 Å in tyrosol, which is also assigned to the role of H in position 3 in the former.

**Fig 3:**
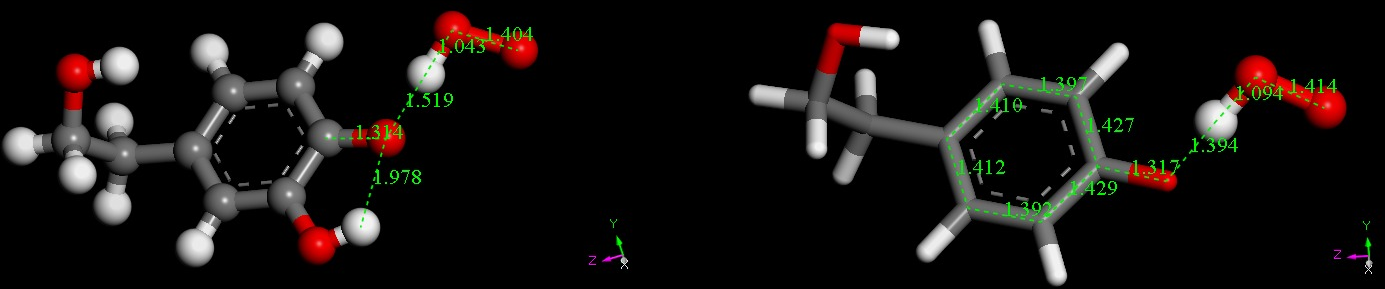
The final state of reaction (1) starting from van der Waals separated hydroxytirosol (left) or tyrosol (right) and O_2_H^−^ (2.60 Å), obtained through geometry minimization.

The product of reaction (**1**) was studied posing the hydroxytyrosol (or tyrosol) radical and HO_2_^-^ anion at van der Waals separation (2.60 Å). The resulting geometry optimizations are depicted in Fig 3, which shows a more efficient role of hydroxytyrosol as a scavenger. Thus, the cleavage of O-H in position 4 is 1.519 Å compared with 1.385 Å in tyrosol, whose H abstraction is weaker. An additional structural feature is seen in Fig 3, the C-O bond at position 4 (1.317 Å), is shorter that in Fig 1 (1.377 Å), that is, after scavenging superoxide there is double bond character in the C-O semiquinone radical at position 4. This also is in agreement with the pattern of double bond conjugation in the aromatic ring, shown in Fig 3. Thus, the two C-C bonds (adjacent to C-O, both involving C4), 1.427 Å and 1.429 Å, are longer than those in the initial state, Fig 2. The remaining bonds 1.392 Å and 1.397 Å are shorter than those involving C4 and longer than those involving C1. Therefore, the aromaticity of the original scavenger has been lost because of scavenging; results from hydroxytyrosol (not shown in Fig 3 for clarity) are consistent with this pattern. The transition state (TS) for reaction (**1**) was calculated and is depicted in S2 Fig. Related thermodynamic data are Delta G = -8.8 kcal/mol for both hydroxytyrosol and tyrosol, whereas the activation energy is 3.4 kcal/mol and 2.0 kcal/mol, respectively. Each transition state shows, as expected theoretically, only one negative frequency in the corresponding infrared spectra, consistent with displacement of H towards O_2_^−•^. For hydroxytyrosol the TS is closer to the product than the reagent: the distances of O(peroxide)—H and O(aromatic)—H are1.143 Å and 1.353 Å, respectively, whereas for tyrosol the abstracted H atom is located midway between reagent and product: O(peroxide)—H = 1.232 Å, O(aromatic)—H = 1.260 Å.

From these results it is concluded that the 2 components of EVOO, tyrosol and hydroxytyrosol, are able to scavenge the superoxide radical, as found experimentally in our CV study described in the next section.

### Cyclovoltammetry

**Fig 4:**
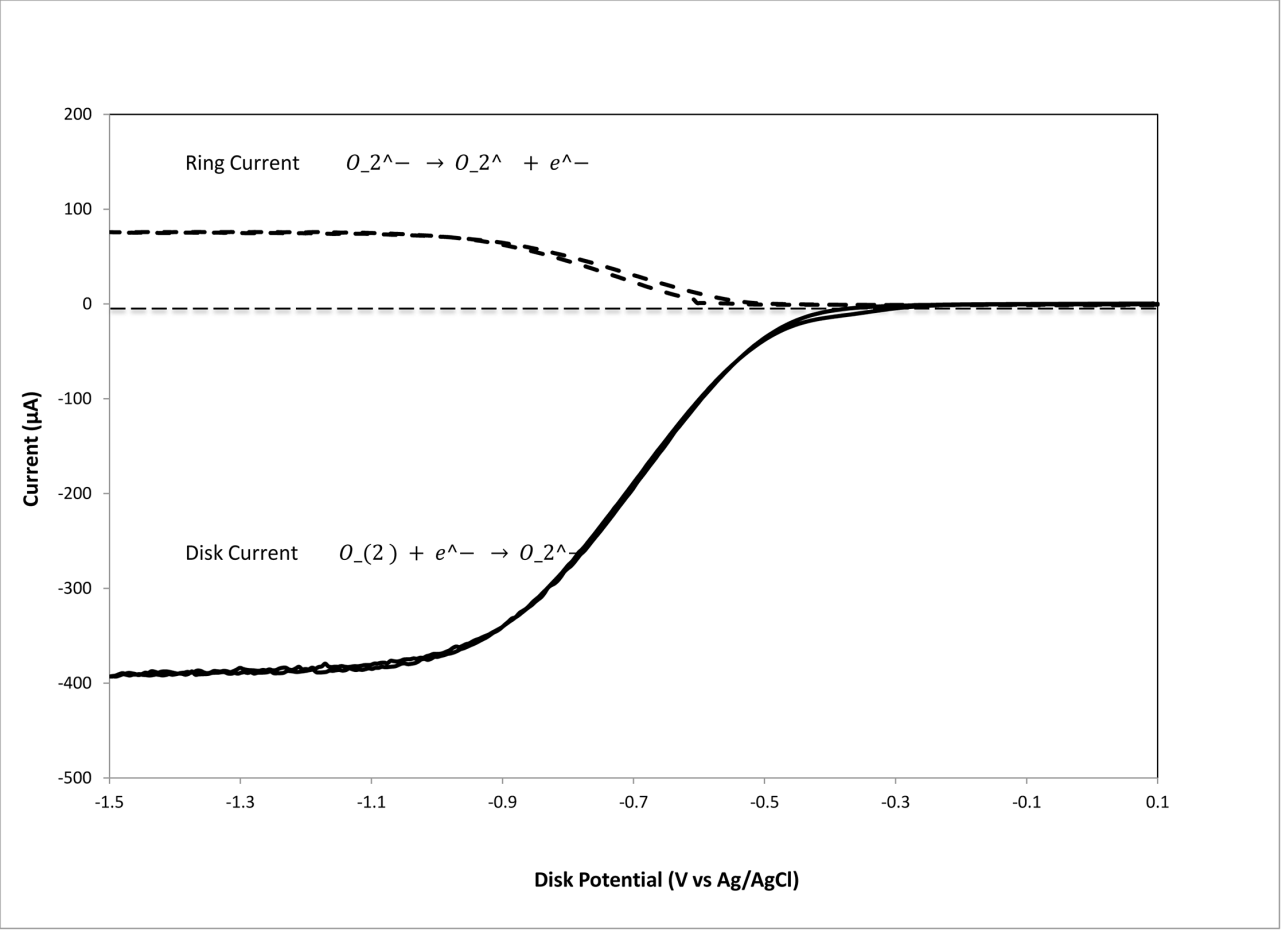
RRDE study of dioxygen in DMSO using gold disk, spinning at 500 rpm, and gold ring electrodes. The potentials were referenced to a Ag/AgCl and the disk was scanned at 25 mV/sec. The coincidence between forward and reverse potential sweep at both electrodes indicates reversibility of this process.

The rotating ring disk electrode (RRDE) allows for a precise study of the kinetics and efficiency of redox reactions. The electrode is rotated at a constant rate and the previously bubbled O_2_ molecules in solution come in contact with the disk. The process at the disc electrode is the reduction of oxygen to superoxide radical (O_2_ + e^-^ → O_2_^-•^), while the reverse reaction occurs at the ring electrode (O_2_^-•^ → O_2_ + e^-^). We focus on the latter reaction (Fig 4).

**Fig 5:**
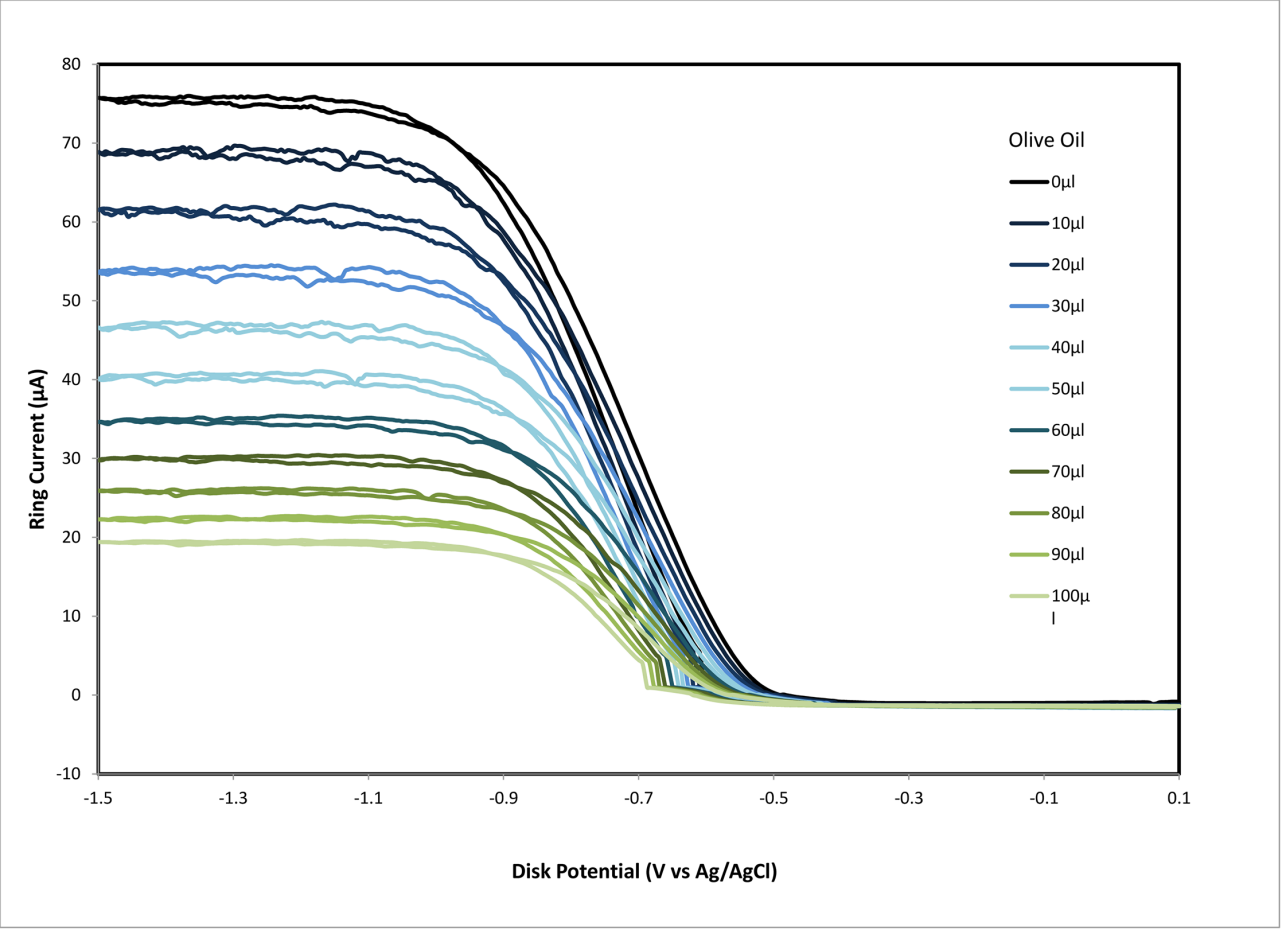
Ring current of the rotating ring disc electrode system showing the decrease in the oxidation current for the oxidation of superoxide ion radical with the addition of olive oil aliquots. The experiment consists of sweeping the disk potential from 0.10 V to -1.50 V and then back to 0.10 V while holding the ring potential at -0.25 volts, sufficiently positive to oxidize superoxide back to O_2_.

The experiment starts at 0.10 V potential on the disc electrode, when the disc electrode has sufficient negative potential and superoxide is generated (Fig 5). The outward flow then sweeps the solution towards the ring (fixed potential -0.25V), allowing the superoxide reverse reaction. This curve corresponds to the CV shown in S1 Fig, as indicated in Materials and Methods section. The method was developed by testing the RRDE’s capacity to measure the superoxide radicals in 50ml of DMSO. Since water destroys superoxide we used dry DMSO.

**Fig 6:**
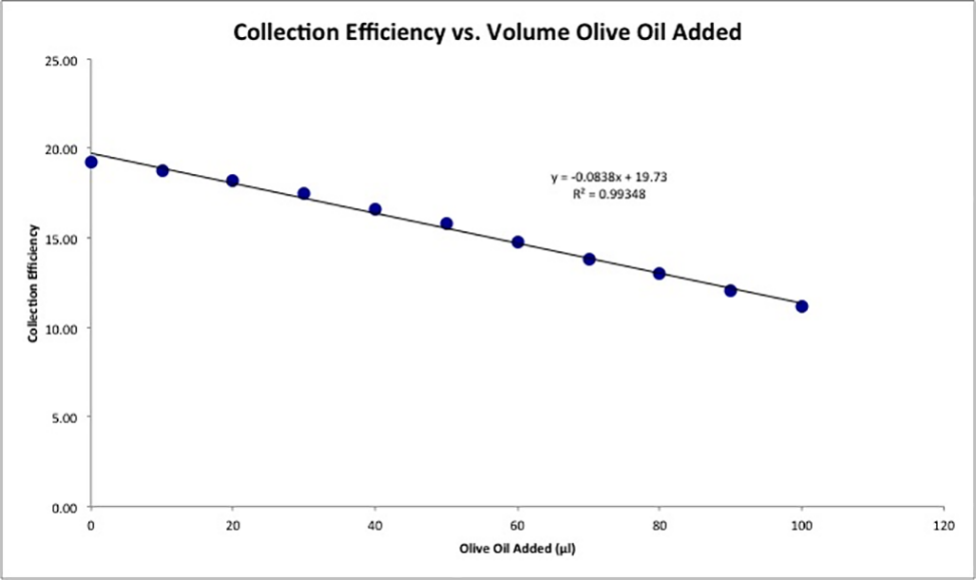
Correlation for superoxide scavenging at the ring electrode due to aliquots of olive oil, as indicated in Fig 5. Collection efficiency is calculated detracting the current at the ring electrode for a given aliquot, at -1.5 V in Fig 5, and the current at time 0, when there was no superoxide yet generated.

After the first run (blank) was completed (Fig 5), addition of an EVOO aliquot (10 μL) results in a significant decrease in the collection efficiency in the RRDE experiment, indicating a removal of the superoxide in the time between generation at the disk and the subsequent oxidation at the ring. Subsequent aliquots decreased superoxide further, showing a linear relationship with the volume of olive oil added over the range of tested volumes. This decrease of superoxide concentration correlates with antioxidants present in EVOO, able to sequester the superoxide radical (Fig 6).

A somewhat related study using a classical CV apparatus, similar to that used to generate S1 Fig, also detects decrease of current intensity of superoxide signal according to increasing amounts of flavonoids added to the electronic cell. In such a system both redox reactions take place at the same eletrode [22]. In contrast, the presence of an additional electrode (the ring electrode) in our system allows us to detect specifically the reaction of interest, namely, the oxidation of superoxide, providing along with more quantitative results regarding its scavenging.

### EPR study

**Fig 7:**
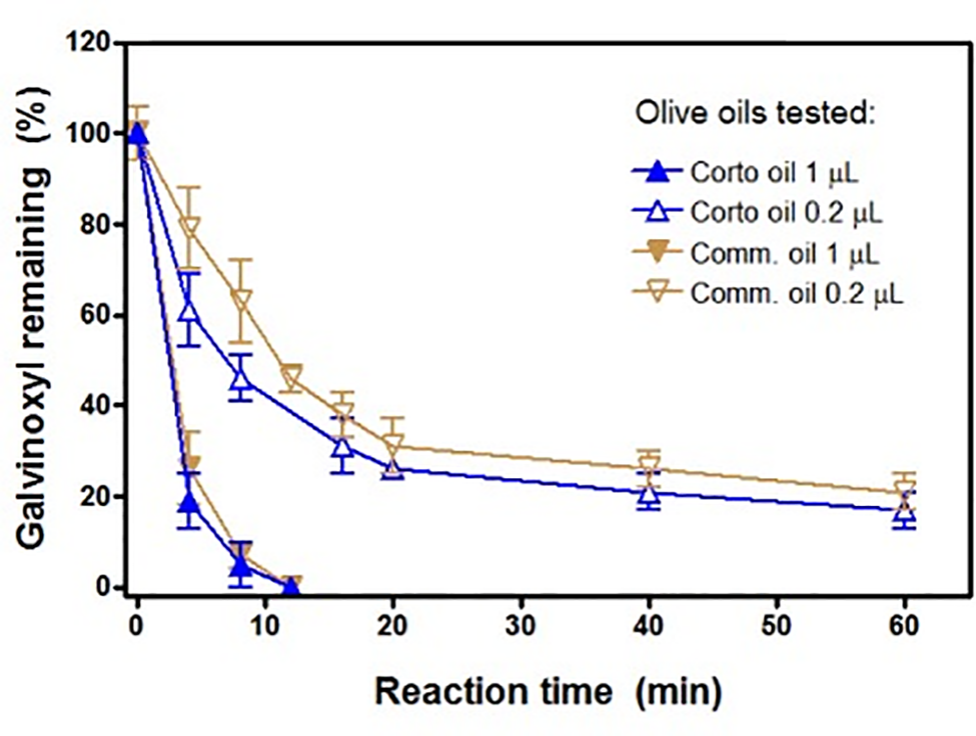
Time-course of galvinoxyl disappearance. Experiments were conducted with 1 μl oil as shown in Fig 8 (1 μl oil to 10 μM galvinoxyl in 100 μl DMSO) and with 0.2 μl oil (1μl oil to 500 μl DMSO) with 10 μM galvinoxyl. Results are the mean ± SD of n = 3 different experiments. Comm. = commercial oil.

From the Fig 7 it is easy to see the disappearance of the stable radical galvinoxyl by oil antioxidants measured directly using EPR spectroscopy. EPR spectra of galvinoxyl (10 μM in 100 μl DMSO) shown before and after addition of commercial EVOO 1 μl at given reaction times. Complete elimination was obtained within 20 min at 25°C. Corresponding antioxidant radical formed in the process was not detected and disappeared rapidly due to dismutation reaction. A representative experiment showing the time-course of the radical scavenging process, less than 10 min for 1 μl of either oil, is also reported (S3 Fig).

### *Caenorhabditis elegans* as a model for oxidative stress and aging

**Fig 8:**
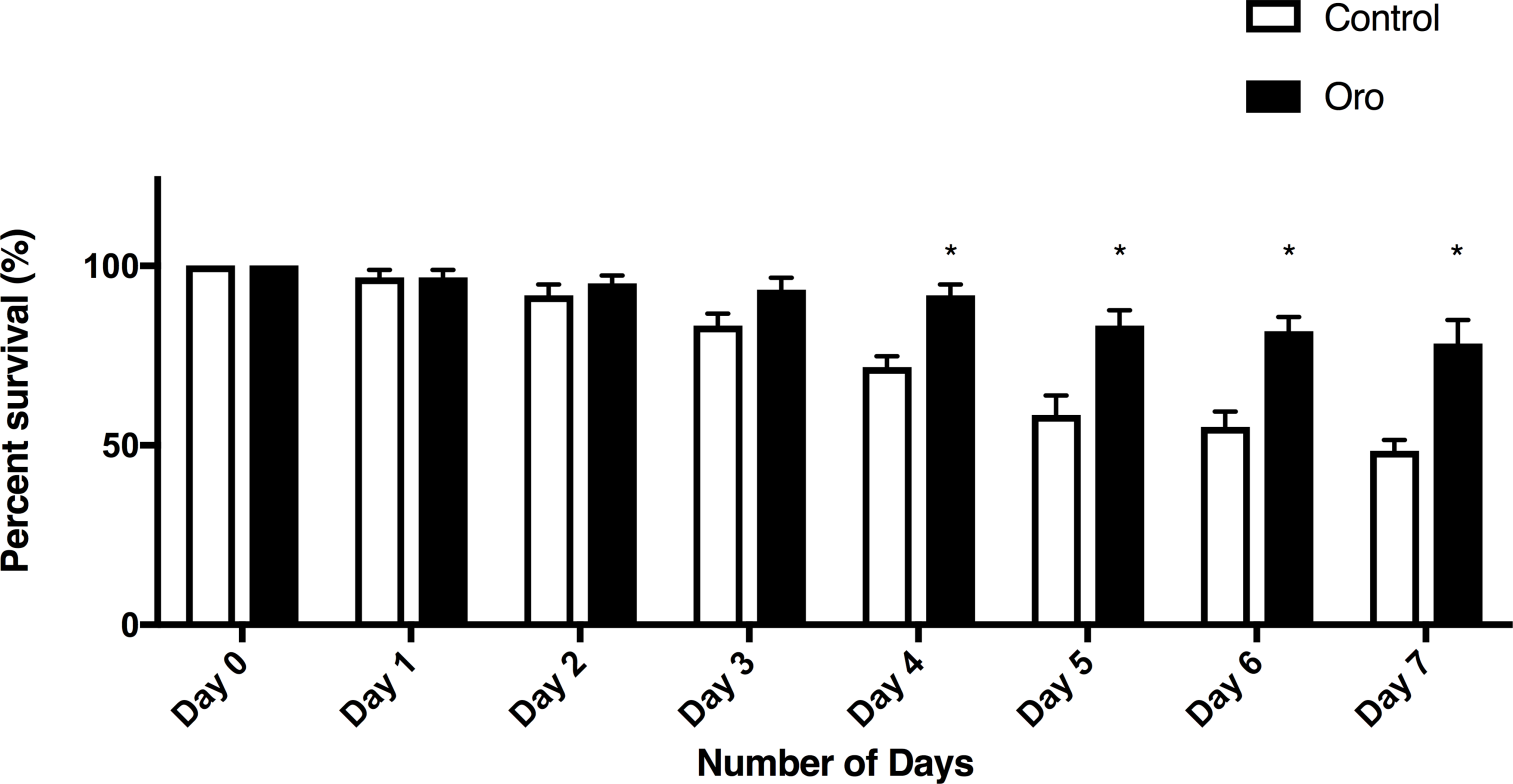
Olive oil enhances survival. L4 nematodes were placed on NGM plates made without or with olive oil, ten nematodes per plate. Survival was scored each day, with surviving worms moved to a fresh treatment plate each day. Two-way ANOVA revealed a significant effect of Oro oil, with greater survival in the presence of the oil than in untreated plates (p<0.001). Bonferroni test for multiple comparisons showed the significant enhancement at days 4–7.

Nematodes grown from the L4 stage onwards on plates containing Oro EVOO survived significantly longer than control nematodes (Fig 8). The survival from days 4 through 7 was enhanced by the Oro EVOO, corresponding to worm ages of 8–11 days. By eleven days post-hatching, nematodes are quite aged [23]. This result is consistent with the well accepted fact that diets including EVOO increase quality of life in Southern European populations and a basis for the popularity of the “Mediterranean diet”.

**Fig 9:**
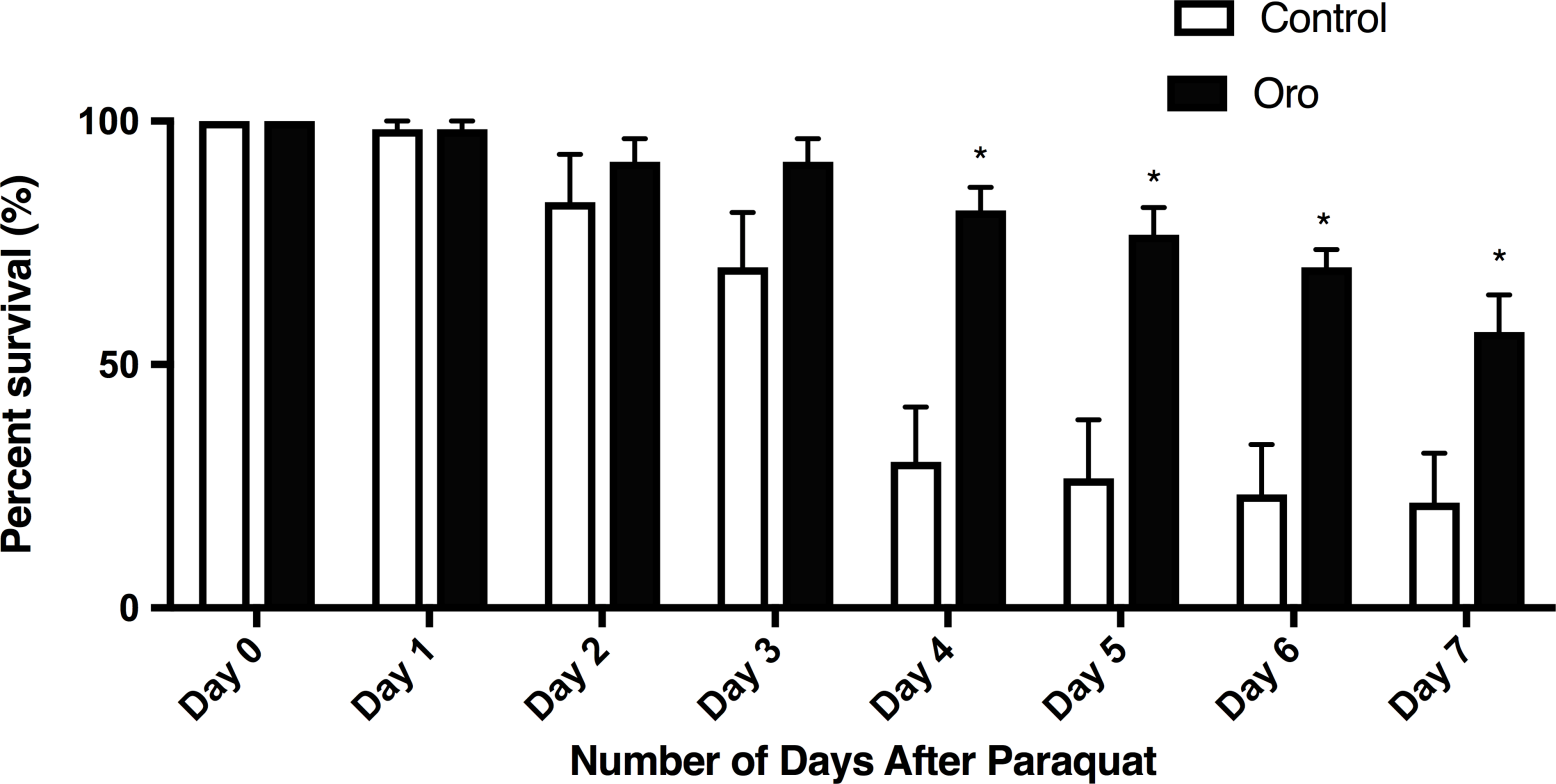
Olive oil enhances survival after paraquat exposure. L4/adult nematodes were placed on NGM plates with 4mM paraquat with or without olive oil in the nematode growth medium. Nematodes were moved to new treated or untreated plates daily for seven days and the percent of 10 worms on each of six replicate plates for each condition was calculated. Data are the average percent survival across the replicate conditions. Two-way ANOVA for treatment and time exposed revealed a significant effect of treatment and of time, as well as a significant interaction effect (p< 0.001). * significantly different from control survival.

The effect of the free radical inducer paraquat (4 mM), on wildtype nematodes is a substantial reduction in survival, Fig 9. It is remarkable that including EVOO in the nematode’s diet the survival after paraquat was significantly increased, demonstrating the health benefits of EVOO, Fig 9. Nematodes were moved to new treated or untreated plates daily for seven days and the percent of 10 worms on each of six replicate plates for each condition was calculated. Data are the average percent survival across the replicate conditions. Two-way ANOVA for treatment and time exposed revealed a significant effect of treatment and of time, as well as a significant interaction effect (p< 0.001). * significantly different from control survival.

#### Effects of EVOO on cell proliferation

**Fig 10:**
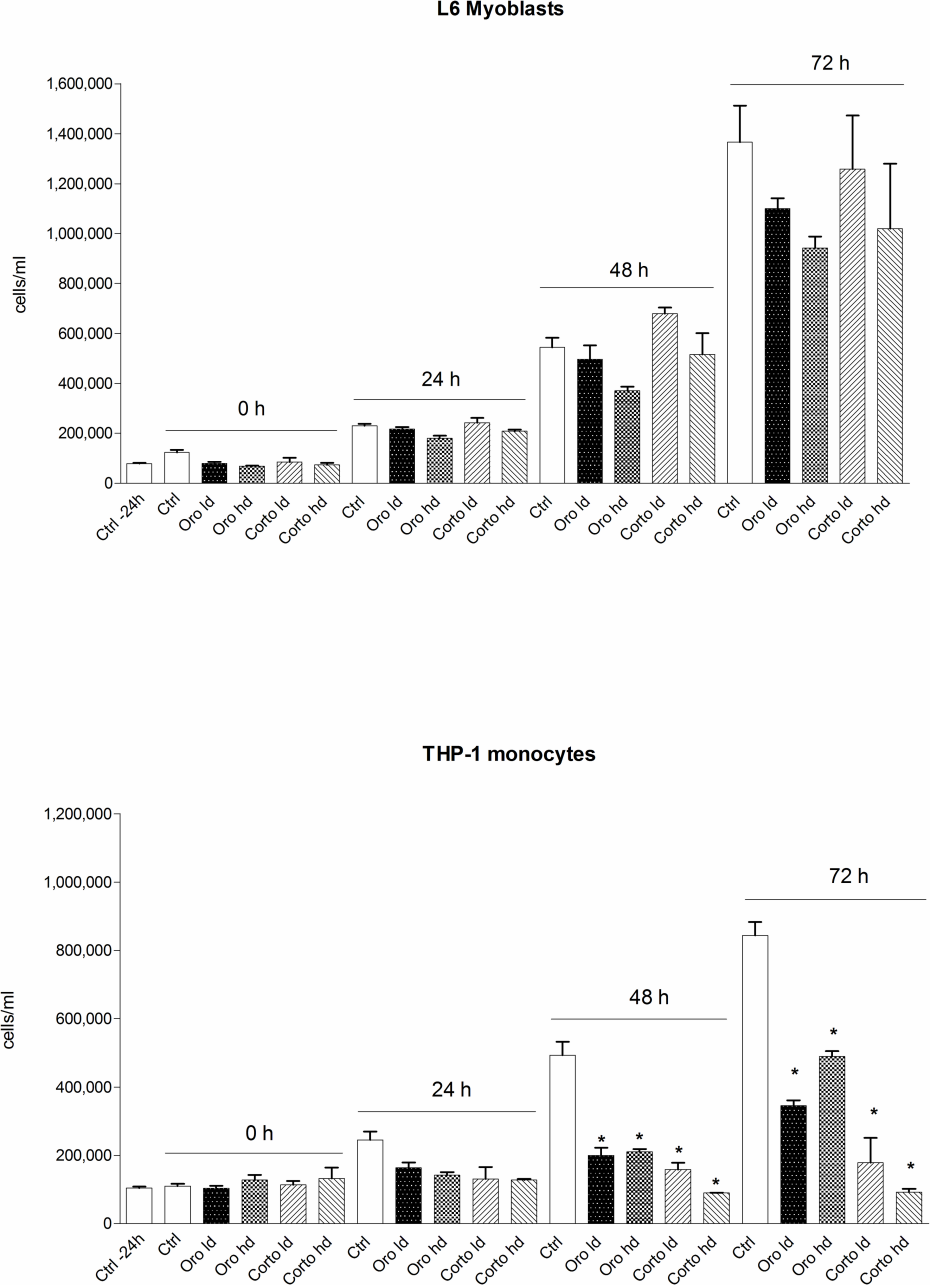
Proliferation curves in the presence of Corto and Oro oils for L-6 myoblasts (upper panel) and THP-1 (lower panel). Cells were seeded in 60 × 15 mm Petri dishes in 4 ml of the respective medium at appropriate density to reach confluence (or maximum density for the THP-1 cells growing in suspension) after 96 h. Cells were stimulated with each oil 24 h from seeding and then counted every 24 h. Results are the mean ± SD of 3 different experiments carried out in duplicate. *p< 0.001, at least, *vs* its own control for both 48 and 72 h.

Cell proliferation was evaluated in both THP-1 monocytes and L6 myoblasts in the presence of Oro and Corto oils. Taken together these results show that both oils did not significantly affect cell proliferation in non tumor L6 myoblast cells. At variance with results in L6 cells, leukemic THP-1 monocytes showed a trend to a decrease of proliferation starting 24 hours after seeding. The inhibitory effect became significant at 48 h and 72 h. In fact, both oils significantly reduced THP-1 proliferation after 48 h and the most striking result was found in cells treated with the high dose of Corto oil. Our results show that both oils were able to prevent proliferation of leukemic monocytes and Corto oil had a stronger inhibitory effect in comparison to Oro oil, whereas in L6 myoblasts the trend to decrease proliferation was never significant in our experimental conditions (Fig 10).

#### Cytotoxicity/proliferation assay (MTT test)

**Fig 11:**
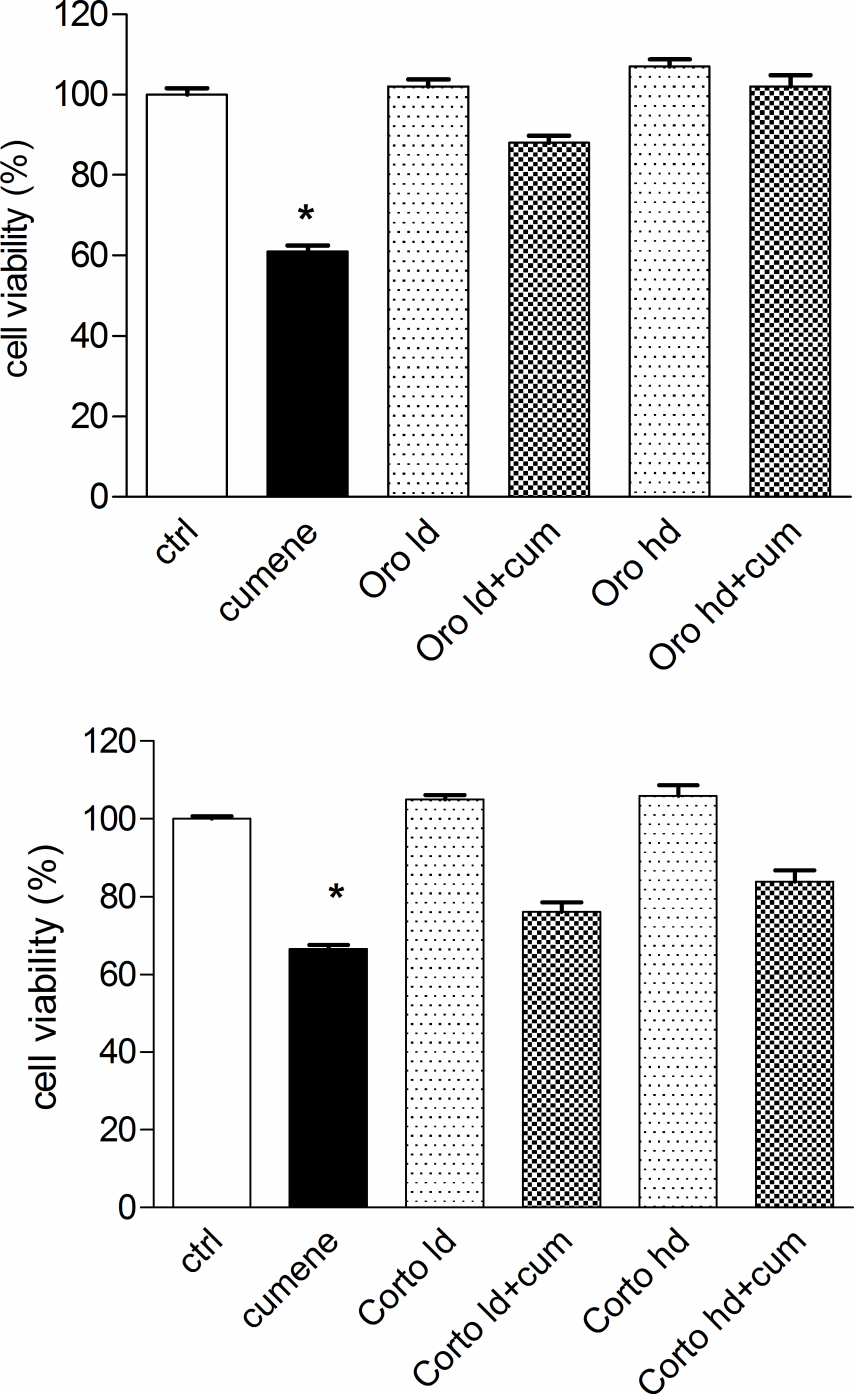
MTT assay carried out with the Corto and Oro olive oils in L6 myoblasts. Cytotoxicity of cumene hydroperoxide 27.5μM in the presence of Corto and Oro oils at the two concentration used throughout the experiments. *p<0.05, at least, *vs* all.

The possible cytotoxicity of the oils together with their protective effect against a radical generator, cumene hydroperoxide, was assessed by MTT assay in L6 myoblasts from rat skeletal muscle. As expected, cumene hydroperoxide alone at the concentration of 27.5 μM significantly reduced cell viability. The concentration of Cumene hydroperoxide 27.5 μM was chosen from our previously published data as a concentration able to induce oxidative stress without major changes in the viability [20]. Our results show that neither Oro oil nor Corto oil, tested at two different concentrations, were toxic when given alone. In fact, no significant difference was detected between Control and Oro or Corto oil after 24 h of the treatment. Interestingly, Oro oil was able to protect L6 myoblasts in a dose-dependent way in the presence of cumene hydroperoxide. In particular, the highest dose of Oro oil was able to completely revert the decreased cell viability induced by the radical generator. Corto oil significantly improved the cell viability of cells treated with oil and cumene hydroperoxide with respect to cumene hydroperoxide alone (p<0.001). It can be concluded that both oils showed a cytoprotective effect against cumene hydroperoxide-induced cytotoxicity (Fig 11).

#### Intracellular ROS determination

**Fig 12:**
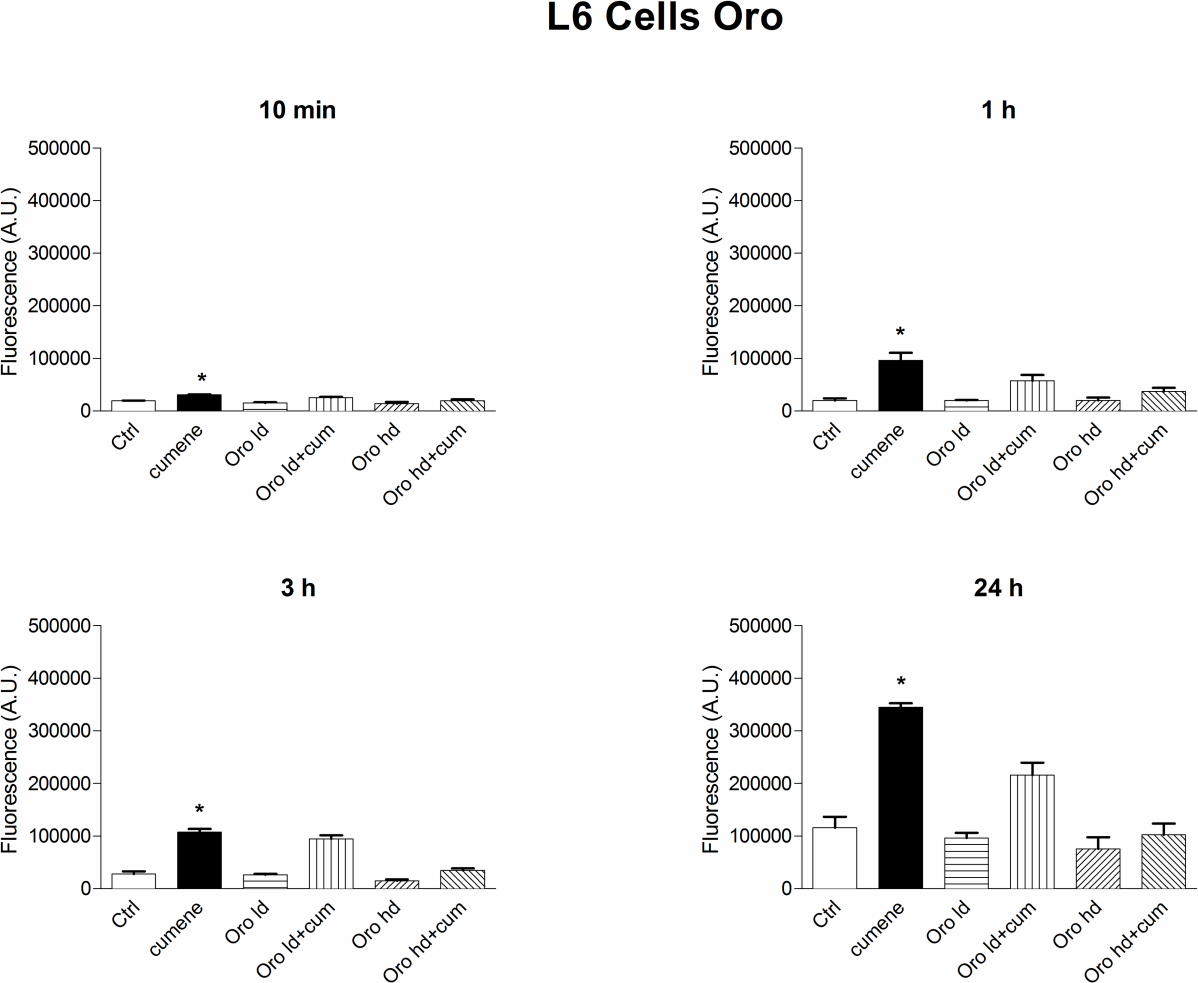
Time course of the capability of Oro oil to protect toward oxidative stress given by Cumene hydroperoxide in L6 myoblasts and THP-1 monocytes. For the L6 myoblasts (upper panel) the concentration of Cumene hydroperoxide was 27.5 μM [18] and 200 μM for the THP-1 monocytes (lower panel). Before addition of cumene hydroperoxide, cells were pre-incubated with oils at 37°C for 10 min, as reported in the Materials and Methods Section. The results are mean of at least two different experiments carried out in duplicate on 12 wells plates. Upper panel: L6 myoblasts with Oro, 10 min * p<0.05 at least, *vs* Ctrl, Oro ld, Oro hd, Cum + Oro hd; 1 h, * p<0.05 at least *vs* Ctrl, Oro ld, Oro hd, cum + Oro ld, 3h * p<0.001 at least.

Since the MTT assay showed that the oils tested protect toward oxidative stress induced by the radical generator cumene hydroperoxide, we studied possible antioxidant activity of Oro and Corto oils using the standard intracellular fluorescent probe assay, based on DCFH2-DA, both in L6 rat myoblasts and THP-1 human leukemic monocytes, as reported in the Materials and Methods. Results obtained were very similar with both oils, we report only data with Oro for the sake of brevity. The cells were preincubated with oils for 10 min and cumene hydroperoxide was then added to induce intracellular ROS formation. Antioxidant activity was tested at the same concentrations used in the proliferation studies in the MTT assay, and at the predefined time points: 10 min, 1 h, 3 h, 24 h and was expressed in arbitrary units of fluorescence, with the lower the fluorescence the better the protection of the cells. Neither of the oils affected the fluorescence by itself. With L6 and THP-1 cells Oro oil was able to protect the cells toward oxidative stress induced by cumene hydroperoxide at all time points (Fig 12).

#### Cell cycle analysis

**Fig 13:**
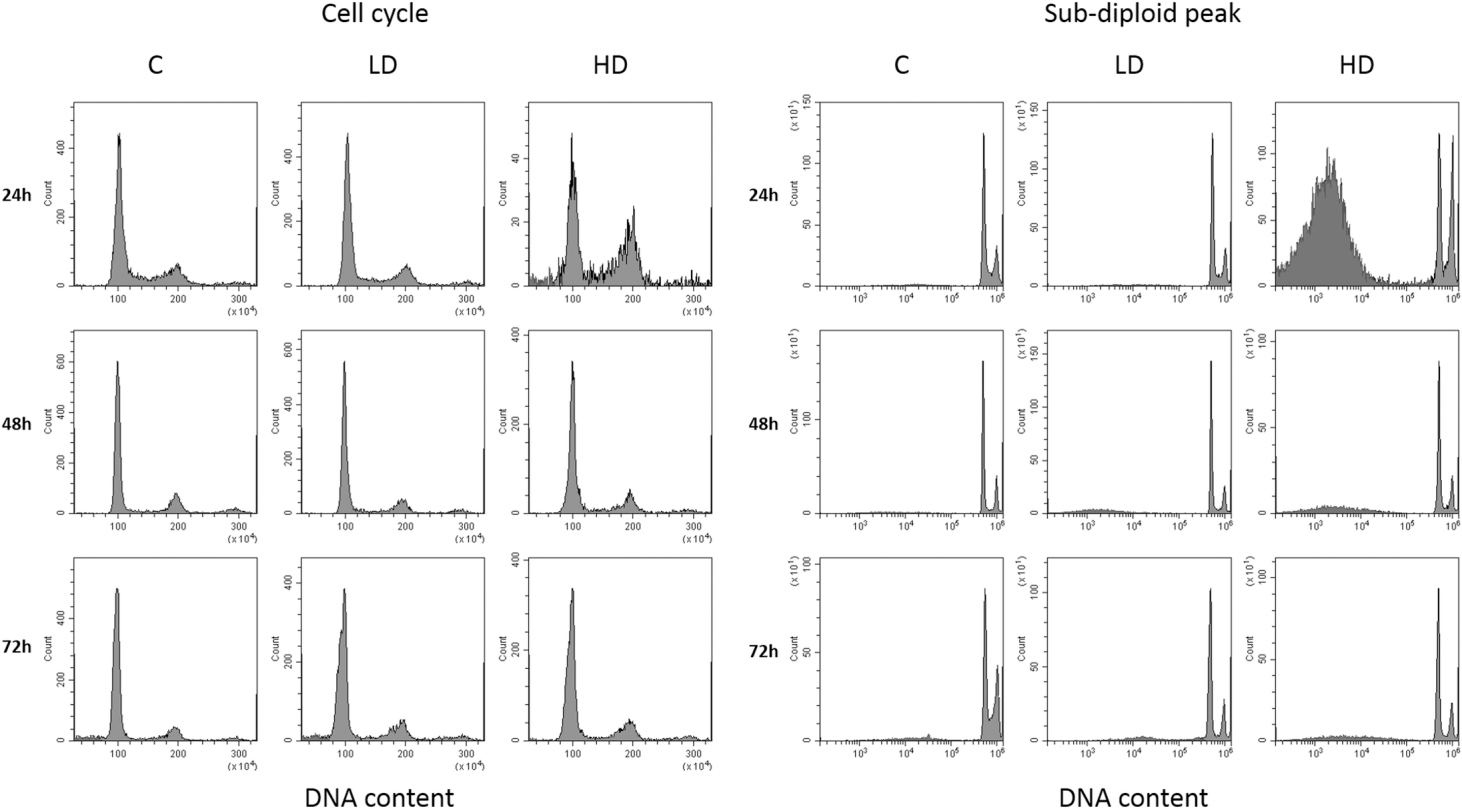
Flow cytometric analysis of DNA after incubation of L-6 myoblasts with Oro oil for 24 h with low dose and high dose as reported under materials and methods. The histogram shows a DNA content distribution of propidium iodide fluorescence of one representative experiment. In the right panel is shown the sub-diploid peak due to DNA apoptosis fragmentation of nuclei.

**Fig 14:**
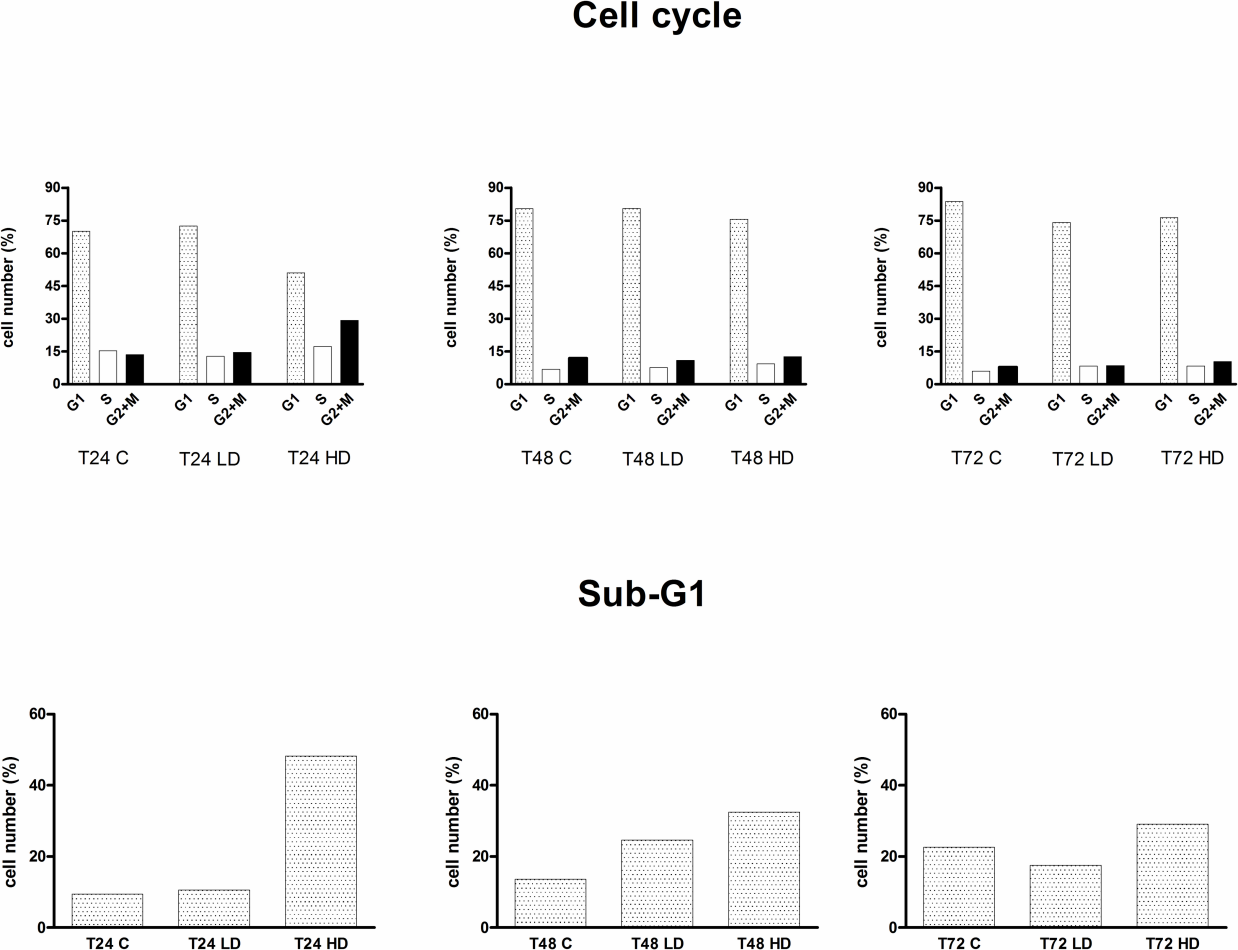
The histogram shows a representative experiment as the percentage of cells cycle phases G1, S and G2/M represent the different phases of the cell cycle determined through the application of electronic markers. In the lower panels is shown the percentage of apoptotic cells found in Sub G1 at different times and doses of Oro oil.

Flow cytometric analysis of cell cycle ditribution was performed on L6 myoblasts and each dose (low and high) was tested on t0-24-48-72 h on chronic treatement. Cell cycle distributions on G1, S and G2/M phases (Fig 13) showed that the oils tested induce a perturbation of cell cycle in particular at 24 h. Analysis of cell distribution (Fig 14) shows that an accumulation of S and G2/M, at the expense of G1 phase, becomes evident at the highest dose. Subdiploid peak, that indicates an apoptosis death induction, increases in a very conspicuous way at 24h of high dose EVOO treatement. This evident early effect it is not observed at longer times probably for a progressive starvation and S phase reduction as a result of the intial cell damage resulting in a recovery from initial cell damage.

It has been recently reported that EVOO slows down the cell cycle in tumor cells with an increase of apoptosis, decrease of the G1 phase and increase of G2. Our data showing the decrease of cell proliferation in THP-1 monocytes in the presence of EVOO oils are in agreement with the data of Coccia et al. [24]. We did not find a significant decrease in L6 cell proliferation, confirmed by the cell cycle analysis and the increase of apoptosis at 24 h. This result could be the equivalent of the slow down response we found in the same L6 myoblast cells when treated with IGF-1 in the presence of T4 [25] a sort of physiological ‘caloric restriction’ aimed at the differentiation process and to cell aging. On the other hand, it is known that antioxidants very often inhibit the proliferation of tumor cells [26–27] leaving unaltered the non–tumor cells. In fact, we found the same differential effects in the cell proliferation of L6 cells and THP-1 monocytes on exposure of antioxidant chalcone derivatives [28]. It is interesting that not only pure compounds but also the EVOO possess this capability, and this may explain several positive aspects of inclusion of olive oil in the Mediterranean diet [29]. This result is confirmed also by additional data resulting from experiments on L6 myoblasts cell cycle carried out with Oro oil. The more striking events were found at 24 h with an increase of G1 and also of the subG1 phase, confirming an arrest of growth and a death of cells for apoptosis that it is found also in the proliferation studies, and the MTT assay where the L6 cells were protected from oxidative stress induced by cumene (p<0.001 with respect to cumene).

### Conclusions

We demonstrate in this study that EVOO experimentally scavenges superoxide radicals very efficiently using a novel two electrode (disc-ring) cyclovoltammetry technique, improving a related one that used only one electrode [22], and this is confirmed by a computational approach for 2 components of olive oil, tyrosol and hydroxythyrosol. EPR analysis of galvinoxyl revealed a full disappearance of this radical in the presence of EVOO, further indicating efficient scavenging ability and consistency with the cyclovoltammetry technique. Further, we report here for the first time a direct positive effect of EVOO on two mammalian cell cultures: leukemic monocytes, a tumor cell line, and myoblasts, a non-tumorous cell line from rat skeletal muscle. We demonstrate using the MTT assay that the L6 cells were protected from oxidative stress induced by cumene hydroperoxide. In addition, both EVOOs studied significantly decreased the proliferation rate of leukemic cells, and kept the proliferation of non-tumorous cells at roughly normal levels. It has been recently reported that EVOO slows down the cell cycle in tumor cells, along with an increase of apoptosis, decrease of the G1 phase and increase of G2 [24], which could account for the reduced proliferation in EVOO-treated cells in our case. Both oils were able to protect the cells against oxidative stress as assessed by ROS determination and by the MTT assay when exposed to radical generator cumene hydroperoxide. We previously showed that hydroxytyrosol reduced oxidative stress caused by cumene hydroperoxide in L-6 myoblasts [30]. In contrast to the effects of EVOO on tumor cell proliferation, the L6 cells were unaffected by the EVOO, but rather had a reduced proliferation likely resulting from the normal process of cell differentiation. Other studies have shown these differential effects of antioxidants [26–27], including our own earlier work on chalcone derivative effects on cell proliferation of L6 cells and THP-1 monocytes [28].

We extended these results to intact multicellular organisms by demonstrating that the ORO EVOO significantly extended *C*. *elegans* lifespan. When the nematodes were exposed to paraquat, a known superoxide generator, EVOO maintained the survival in 50–60% of nematodes at the level of untreated nematodes, whereas paraquat-exposed nematodes not treated with EVOO exhibited a dramatic decrease in survival. Stress responses, including oxidative stressors, influence aging and lifespan *C*. *elegans*. One recent study identified SKN-1/Nrf as an important regulator of these processes [27], which may be a target of the protective effects of EVOO; our future work can address this mechanism. In conclusion, our work demonstrates that using EVOO has significant beneficial effects as measured on cell culture growth and *C*. *elegans* life cycle. Our results support the extensive health benefits of systematically using EVOO in human diets.

## Supporting information

S1 Fig A cyclic voltammogram, taken after bubbling O_2_ in DMSO dry solution, of the oxygen/superoxide redox couple showing the potentials at which reduction and oxidation occur. Minimum corresponds to O_2_ reduction; maximum to superoxide anion oxidation. Initial potential = 0.10V.
(TIF)

S2 Fig Transition state of reaction (1) for hydroxytirosol (left) or tyrosol (right). (TIF)

S3 Fig Reduction of the stable radical galvinoxyl by EVO oil antioxidants measured directly using EPR spectroscopy. EPR spectra of galvinoxyl (10 μM in 100 μl DMSO) shown before and after addition of oil (1 μl), at given reaction times. Complete elimination of galvinoxyl obtained within 20 min at 25°C. Corresponding antioxidant radicals formed in the process, not detected, which disappear rapidly due to dismutation reaction. a) Galvinoxyl radical, b) olive oil, 2 min; c) olive oil, 4 min; d) olive oil; 8 min; e) olive oil, 18 min.
(TIF)
