# Supplementary figures and images for: Protection by extra virgin olive oil against oxidative stress in vitro and in vivo. Chemical and biological studies on the health benefits due to a major component of the Mediterranean diet
Source: PLoS One. 2017 Dec 28;12(12):e0189341. doi: 10.1371/journal.pone.0189341 (PMC5746230; doi:10.1371/journal.pone.0189341)

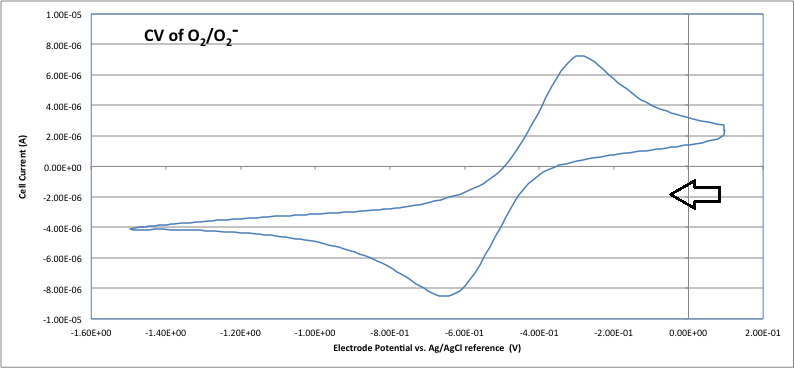

Supplement: S1 Fig — Minimum corresponds to O2 reduction; maximum to superoxide anion oxidation. Initial potential = 0.10V. (TIF) [file pone.0189341.s001.tif]

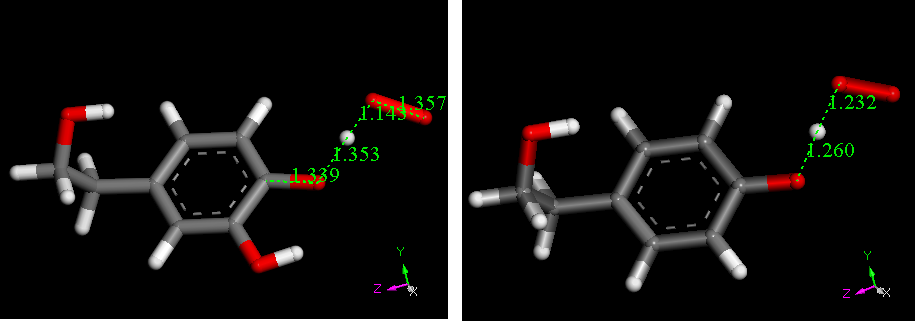

Supplement: S2 Fig — (TIF) [file pone.0189341.s002.tif]

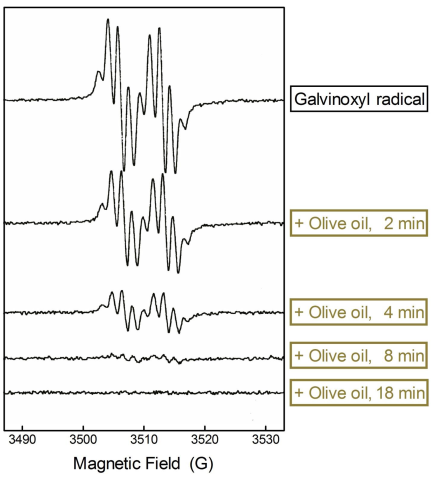

Supplement: S3 Fig — EPR spectra of galvinoxyl (10 μM in 100 μl DMSO) shown before and after addition of oil (1 μl), at given reaction times. Complete elimination of galvinoxyl obtained within 20 min at 25°C. Corresponding antioxidant radicals formed in the process, not detected, which disappear rapidly due to dismutation reaction. a) Galvinoxyl radical, b) olive oil, 2 min; c) olive oil, 4 min; d) olive oil; 8 min; e) olive oil, 18 min. (TIF) [file pone.0189341.s003.tif]
